# Foreign body causing perforation of the appendix in an African boy

**DOI:** 10.4314/pamj.v5i1.56193

**Published:** 2010-04-24

**Authors:** Peter Hulme

**Affiliations:** 1 Trafford General Hospital, Moorside Road, England

**Keywords:** Appendicitis, foreign body, Africa, paediatrics, perforation

## Abstract

Foreign bodies in the appendix are a rare but well described clinical entity and may cause perforation. Presented here is the case of a 13yr old Ugandan boy who had features of acute appendicitis, was sent for appendicectomy and during the operation was found to have perforation of the appendix due to a seed. The boy was treated with broad spectrum antibiotics and made an uneventful recovery.

## Background

Foreign bodies in the appendix are well recognised but rarely found. The author is not aware of any other reported cases of a foreign body causing perforation of the appendix in a child in Africa. Presented here is the case of a 13yr old Ugandan boy with abdominal pain who was found to have a perforated appendix with a seed sitting in the perforation.

## Patient and case report

A 13 years old male presented to hospital with intermittent abdominal pains for one month associated with watery diarrhoea and mild fever. He had recently taken a course of albendazole. Examination revealed mild tenderness in the right iliac fossa (RIF) and right upper abdomen. A stool sample was negative for parasites and oocysts. It was thought that he was having acute appendicitis and was admitted to the surgical ward. He was treated with oral metronidazole and ciprofloxacin but after two days his symptoms improved and he was discharged home.

Eighteen days later he re-attended surgical clinic complaining of right sided abdominal pain. Again he was tender in the RIF but there was no guarding or signs of peritonism. He was readmitted to the surgical ward and again put on oral metronidazole and ciprofloxacin. His Hb was 8.6g/dl.

**Figure 1:**
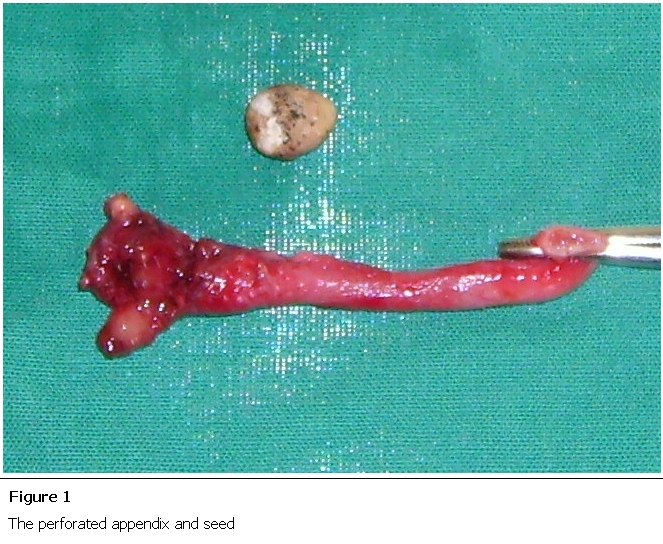
The perforated appendix and seed

Three days after admission the patient was sent for appendicectomy. On the day of the operation his abdomen was soft with mild RIF tenderness and active bowel sounds. There were no features of peritonism. The appendix was located via a grid iron incision and was found to be laying retrocaecally. It was inflamed with a distal perforation and an oval shaped, 13x8 mm seed was seen to be partially protruding from the perforation (Figure 1). No pus was seen in the abdomen. Peritoneal lavage was performed with warmed 0.9% saline. The patient was put on X-Pen and gentamicin 160 mg with IV fluids. The patient made an uneventful recovery and was discharged home three days post-op in a stable condition. The patient didn’t recognise the seed nor could he recall eating it. The seed was sent to Makerere University and the photograph showed to local people but it was not able to be identified.

## Discussion

Abdominal pain in children is a common problem seen by doctors all over the world. Pain in the abdomen has a broad differential diagnosis encompassing relatively benign conditions such as urinary tract infection or constipation to life threatening emergencies such abdominal perforation [1]. Appendicitis is the most commonly performed emergency abdominal surgery and the leading cause of acute paediatric surgical admission yet determining which patients have appendicitis is difficult [2,3]. Various scoring systems based on clinical signs have been shown to be of use in identifying patients at high risk of appendicitis [4].

Foreign bodies in the appendix are very uncommon with one study showing a frequency of 0.005% in 13,228 patients [4]. The literature has described many causes of foreign bodies in the appendix such as seeds, needles, tongue studs, parasitic worms, bullets and dental drill pieces [5,6]. Long, thin, sharp and pointed items are thought to be most likely to cause perforation after ingestion whilst seeds are thought to present a medium risk of perforation [5]. Appendicitis can even occur after appendicectomy, a phenomenon known as ‘stump appendicitis’ so surgeons still need to consider a diagnosis of appendicitis in patients who have had their appendix removed [7].

## Conclusion

Acute appendicitis is a surgical emergency and delay in performing appendicectomy increases the risk of perforation. Perforation of the appendix due to a foreign body can occur without classical signs and symptoms of perforation. Surgeons all over the world should be aware of the potential operative findings of a foreign body and be able to deal with them appropriately.

## Consent

The patient and his family gave consent for the article to be written.

## Competing interests

The author declared they have no competing interests.
